# Investigating the role of LSD2 as an epigenetic regulator in Ewing sarcoma

**DOI:** 10.18632/oncotarget.26988

**Published:** 2019-06-11

**Authors:** Priyal O. Patel, Kathleen I. Pishas, Cenny Taslim, Julia Selich-Anderson, Emily R. Theisen, Stephen L. Lessnick

**Affiliations:** ^1^The Division of Pediatric Hematology, Oncology & Blood and Marrow Transplant, Department of Pediatrics, The Ohio State University, Columbus, OH, USA; ^2^Center for Childhood Cancer and Blood Diseases, The Research Institute at Nationwide Children’s Hospital, Columbus, OH, USA

**Keywords:** LSD2/KDM1B, Ewing sarcoma, SP-2509, LSD1/KDM1A

## Abstract

Ewing sarcoma is the second most common solid bone malignancy diagnosed in pediatric and young adolescent populations. Despite aggressive multi-modal treatment strategies, 5-year event-free survival remains at 75% for patients with localized disease and 20% for patients with metastases. Thus, the need for novel therapeutic options is imperative. Recent studies have focused on epigenetic misregulation in Ewing sarcoma development and potential new oncotargets for treatment. This project focused on the study of LSD2, a flavin-dependent histone demethylase found to be overexpressed in numerous cancers. We previously demonstrated that Ewing sarcoma cell lines are extremely susceptible to small molecule LSD1 blockade with SP-2509. Drug sensitivity correlated with the degree of LSD2 induction following treatment. As such, the purpose of this study was to determine the role of LSD2 in the epigenetic regulation of Ewing sarcoma, characterize genes regulated by LSD2, and examine the impact of SP-2509 drug treatment on LSD2 gene regulation. Genetic depletion (shRNA) of LSD2 significantly impaired oncogenic transformation with only a modest impact on proliferation. Transcriptional analysis of Ewing sarcoma cells following LSD2knockdown revealed modulation of genes primarily involved in metabolic regulation and nervous system development. Gene set enrichment analysis showed that SP-2509 does not impact LSD2 targeted genes. Although there are currently no small molecule agents that specifically target LSD2, our results support further investigations into agents that can inhibit this histone demethylase as a possible treatment for Ewing sarcoma.

## INTRODUCTION

Ewing sarcoma is the second most common solid bone malignancy diagnosed in pediatric and young adolescent populations [1]. Genetically, it is characterized by the presence of a somatic translocation between the 5’ portion of *EWSR1* on chromosome 22 with the 3’ portion of an ETS transcription factor [2]. 85% of all translocations in Ewing sarcoma consist of the reciprocal chromosomal translocation (11;22) (q24;q12), which encodes EWS/FLI, the distinguishing oncogenic driver required for Ewing sarcomagenesis [3]. Understanding how EWS/FLI functions may uncover potential targets for novel therapeutic approaches.

Previous research has focused on defining key EWS/FLI protein interactions that regulate the expression of tumor suppressors and oncogenes. One such interaction is between EWS/FLI and the Nucleosome Remodeling and Deacetylase (NuRD) co-repressor complex [4]. Lysine Specific Demethylase 1 (LSD1/KDM1A) is often associated with the NuRD complex and in Ewing sarcoma is required to mediate the repressive function of EWS/FLI. LSD1 regulates EWS/ETS-mediated transcriptional dysregulation of both EWS/ETS-repressed and -activated genes [4, 5]. It is a flavin-adenine dependent (FAD) amine oxidase that regulates chromatin function through histone demethylation [6, 7]. LSD1 is overexpressed in several malignancies, including Ewing sarcoma, and overexpression is associated with poor prognosis [3, 8, 9]. LSD1 is crucial for Ewing sarcoma cell proliferation and oncogenic transformation both *in vitro* and *in vivo* [3, 5, 10].

The critical role of LSD1 in epigenetic regulation has encouraged the development of several targeted small molecule agents including GSK2879552, INCB059872, IMG-7289, and ORY-2001 [3, 7, 10, 11]. Previous data has shown that treatment of Ewing sarcoma cell lines with the reversible non-competitive LSD1-inhibitor SP-2509 resulted in the reversal of the EWS/FLI-driven transcriptional signature, the triggering of unfolded protein response-mediated apoptosis, and led to single agent tumor regression *in vivo* [3]. Interestingly, mRNA and protein induction of the mammalian homolog of LSD1, LSD2 (KDM1B), following SP-2509 treatment strongly correlated with drug sensitivity [3, 10]. Genetic depletion (shRNA) of LSD2 significantly reduced the efficacy of SP-2509 only in Ewing sarcoma cells highly sensitive to the drug, implying that LSD2 influences SP-2509 cytotoxicity [3]. These data suggest that SP-2509 affects both LSD1 and LSD2. However, the contribution of each to the Ewing sarcoma phenotype remains unknown.

LSD2, also known as KDM1B, shares 31% sequence similarity with LSD1 [3, 10]. Despite sharing sequence and structural similarities, LSD1 and LSD2 are thought to function in distinct chromatin-remodeling complexes [12]. Both contain a FAD coenzyme-binding motif, a SWIRM domain, and a carboxyl-terminal amine oxidase domain [10, 13]. However, the amino-terminus of LSD1 is disordered, while the amino-terminus of LSD2 contains a zinc finger domain [12, 13]. The zinc-finger domain in LSD2 mediates interactions with nucleosomal DNA and other chromatin-associated proteins [12]. Additionally, LSD1 contains a 100 amino-acid tower domain that functions as the binding site for the CoREST protein required for LSD1 stability and activity *in vivo* and *in vitro* [12]. In contrast, the tower domain is absent in LSD2 [10, 13, 14]. Instead, LSD2 interacts with the linker region of glyoxylate reductase 1 homolog (GLYR1/NPAC), a nuclear protein which helps regulate the enzymatic activity of LSD2 [13, 14]. Functionally, both LSD1 and LSD2 have H3K4me1/2-specific histone demethylase activity [6, 12]. The majority of LSD1 is located at promoters, while LSD2 typically binds downstream of promoter regions, peaking towards the 3’ end of the target gene [13]. LSD1 functions as a co-activator and co-repressor, while LSD2 is associated with transcribed coding regions in genes and is important for optimal gene transcription [10, 13, 15].

LSD2 has been noted to be overexpressed in several malignancies such as colorectal adenocarcinoma, small cell lung cancer and breast cancer [16, 17, 18]. Recent studies with breast cancer cell lines showed that overexpression of LSD2 led to increased cell proliferation and oncogenic transformation, but decreased cellular motility and invasion [15]. Similarly, in small cell lung cancer, suppression of LSD2 decreased proliferation by indirectly regulating the expression of the tumor suppressor gene *TFPI-2* [18]. However, LSD2’s role in Ewing sarcomagenesis is not well understood. Furthermore, it is unclear what isoforms of LSD2 are expressed in Ewing sarcoma and what impact different isoforms have on tumorigenesis. In addition, although our previous SP-2509 data has suggested a link between LSD2 expression and drug cytotoxicity, it is unclear if this occurs through LSD2-mediated transcriptional regulation [3].

In this study, we designed a series of experiments to investigate the role of LSD2 in the epigenetic misregulation of Ewing sarcoma. We utilized RNA sequencing and pathway analysis to characterize genes regulated by LSD2, and used gene set enrichment analysis to study how drug treatment with SP-2509 impacts LSD2 gene regulation. We hypothesized that LSD2 is an epigenetic enzyme critical for cell proliferation and oncogenic transformation of Ewing sarcoma cells, and predicted that SP-2509 inhibits not only LSD1, but also LSD2.

## RESULTS

### A673 cells express two LSD2 isoforms

**Figure 1:**
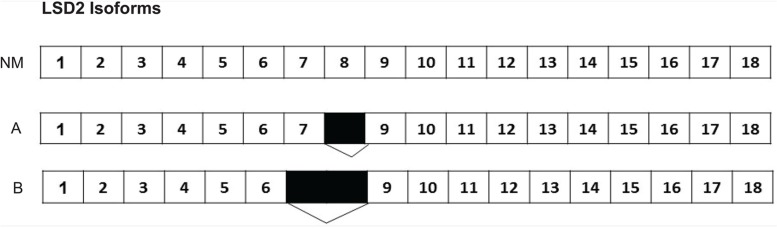
Two LSD2 isoforms identified in A673 cells. The most widely studied LSD2 isoform (NM_153042.3) in the NCBI database has 18 exons. The first isoform isolated from A673 cells had a total of 17 exons, with exon 8 missing. The second isoform of LSD2 had only 16 exons, with exons 7 and 8 missing. Numbers indicate LSD2 exon number with black boxes denoting deleted regions.

According to the National Center for Biotechnology Information (NCBI) database, 11 isoforms of LSD2 exist with the most commonly reported one containing 18 exons (NCBI Reference Sequence: NM_153042.3) [19]. No studies have directly assessed isoform expression in Ewing sarcoma cell lines. Sequencing analysis of A673 cells revealed two isoforms of LSD2. The first isoform we isolated had a total of 17 exons, with exon 8 missing. The second isoform of LSD2 had only 16 exons, with 7 and 8 missing (Figure 1). Our data on the isoforms is limited to A673 cells and the function of different LSD2 isoforms remains broadly unexplored.

### Knockdown of LSD2 impacts both proliferation and oncogenic transformation

**Figure 2:**
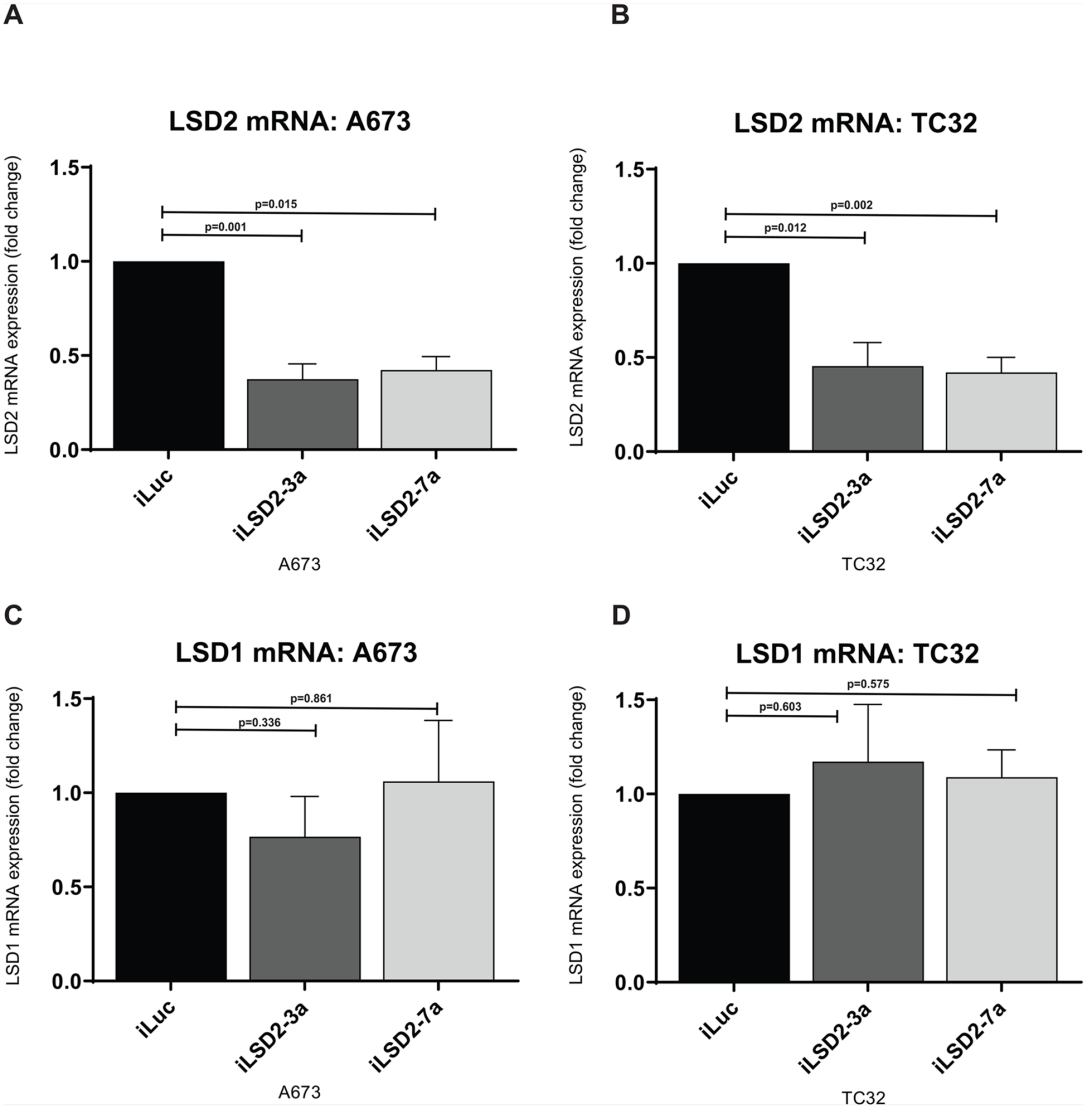
LSD2 mRNA expression decreased after retroviral infection with shRNAs. **(A/B)** qRT-PCR analysis of LSD2 mRNA expression in A673 and TC32 cells following retroviral infection with either LSD2 shRNA (iLSD2-3a/iLSD2-7a) or control (iLuc) constructs. Cells collected 3 days post puromycin selection. Data represents mean expression ± SEM from three independent experiments. **(C/D)** qRT-PCR analysis of LSD1 mRNA expression from cells described as above.

To ascertain whether LSD2 is required for Ewing sarcomagenesis, we assessed the proliferative and anchorage independent growth of Ewing sarcoma cell lines following shRNA-mediated knockdown of LSD2. Successful knockdown of endogenous LSD2 mRNA and protein expression was achieved through retroviral infection of A673 and TC32 Ewing sarcoma cells with two distinct LSD2 shRNAs (iLSD2-3a, iLSD2-7a). The shRNAs each targeted both LSD2 isoforms identified in A673 cells by targeting the 3’UTR of the mRNA. Compared to a negative control targeting luciferase (iLuc), a 63% (iLSD2-3a; p-value: 0.001) and 58% (iLSD2-7a; p-value: 0.015) decrease in LSD2 mRNA expression was observed in A673 cells following 3 days of puromycin selection (Figure 2A). In TC32 cells, a 54% and 58% reduction in mRNA expression was achieved after retroviral infection with iLSD2-3a (p-value: 0.012) and iLSD2-7a (p-value: 0.002), respectively (Figure 2B). LSD1 mRNA expression was not significantly affected by LSD2 knockdown in the two cell lines (<25% change) (Figure 2C-2D).

**Figure 3:**
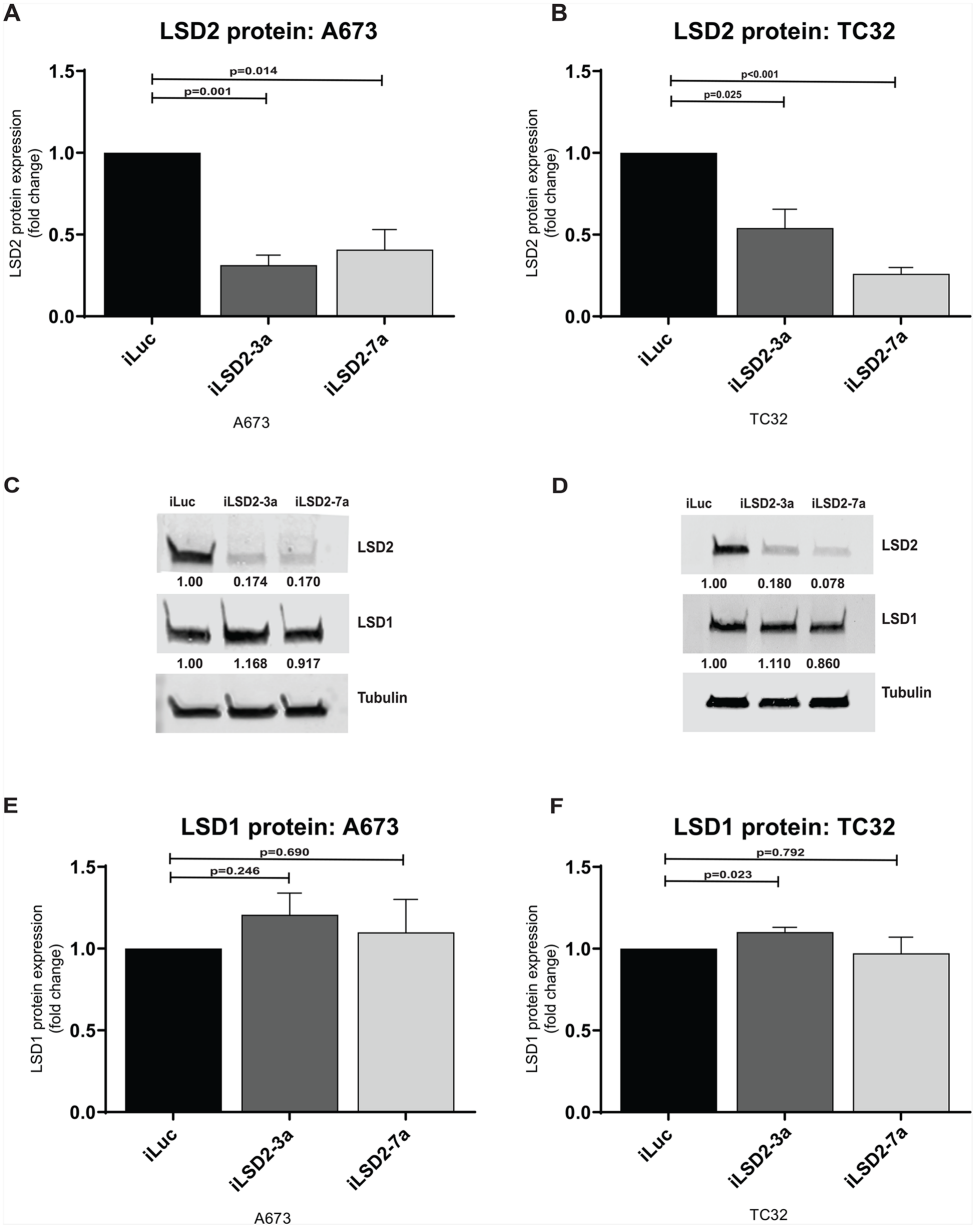
LSD2 protein expression following retroviral knockdown. **(A/B)** Densitometry quantification of LSD2 protein levels in A673 and TC32 cells following retroviral infection with either LSD2 shRNA (iLSD2-3a/iLSD2-7a) or control (iLuc) constructs. Cells collected 3 days post puromycin selection. Data represents mean fold change ± SEM compared to iLuc control from 3 independent experiments. **(C/D)** Representative western blot images of LSD2, LSD1 and α-tubulin (loading control) protein levels from cells described as above. **(E/F)** Densitometry quantification of LSD1 protein levels from A673 and TC32 cells treated as above.

Protein expression of LSD2 was also reduced after retroviral infection. For A673 cells, a 69% and 59% reduction in protein expression was achieved for iLSD2-3a (p-value: 0.001) and iLSD2-7a (p-value: 0.014), respectively (Figure 3A). In TC32 cells, a 46% and 74% reduction was noted in the cells treated with iLSD2-3a (p-value: 0.025) and iLSD2-7a (p-value<0.001), respectively (Figure 3B). LSD1 protein expression was minimally impacted by endogenous knockdown of LSD2 in the two cell lines (<25 % change) (Figure 3C-3F).

**Figure 4:**
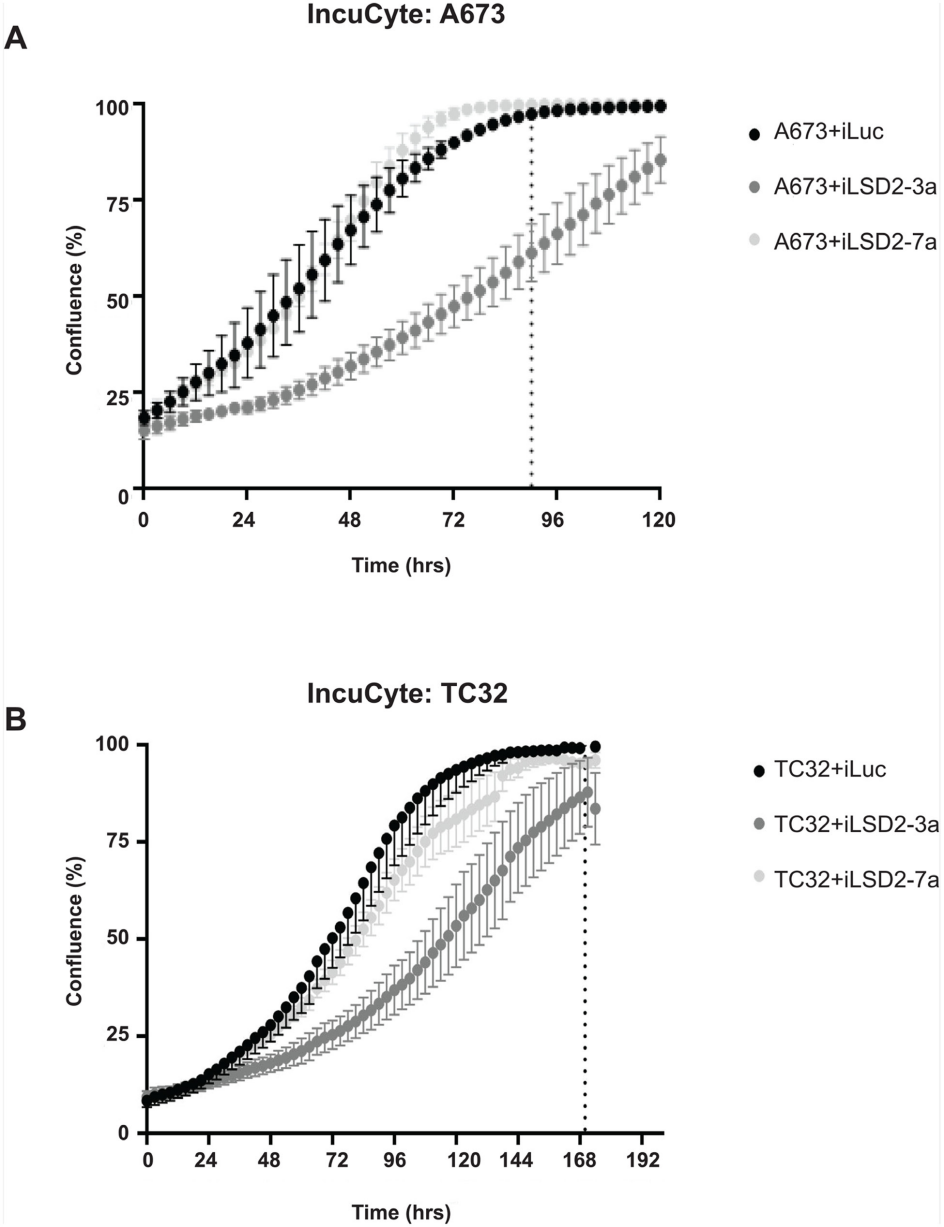
LSD2 knockdown and its impact on Ewing sarcoma cellular proliferation. IncuCyte proliferation analysis of A673 **(A)** and TC32 **(B)** cells following retroviral infection with either LSD2 shRNA (iLSD2-3a/iLSD2-7a) or control (iLuc) constructs. Phase contrast images taken every three hours. Data represents mean confluence ± SEM from 3 independent experiments. Dashed line denotes time taken for iLuc control cells to reach 100% confluency.

To elucidate whether LSD2 is required for Ewing sarcoma cell proliferation, live cell IncuCyte imaging of cells following LSD2 knockdown was performed. Although, iLSD2-7a had no significant effect on cell proliferation for both cells lines compared to iLuc control, iLSD2-3a modestly impaired the growth of A673 and TC32 cells. After 90hrs of cellular growth, the confluency of A673 iLuc cells was 100% whereas cells with LSD2 knockdown (iLSD2-3a) were only at 59% (p-value <0.001) (Figure 4A). Similar results were observed for TC32 cells, with 100% and 86% confluency respectively, 170hrs post seeding (p-value <0.001; Figure 4B). The reason for the discrepancy between the data for the two shRNA is unclear but may be because of the following: 1) a specific fold decrease in LSD2 knockdown may be required to significantly impact proliferation; 2) the two shRNAs target the 3’UTR region of LSD2 at different locations and may have different off target effects; 3) they may impact various LSD2 isoforms differently, which may affect proliferation. It must be noted that we previously published that both iLSD2-3a and iLSD2-7a impaired the proliferative growth of both A673 and EWS-502 cells when greater than 75% knockdown was achieved [3]. Our results suggest that knockdown of LSD2 with iLSD2-3a did modestly impact proliferation, while knockdown with the second hairpin did not.

**Figure 5:**
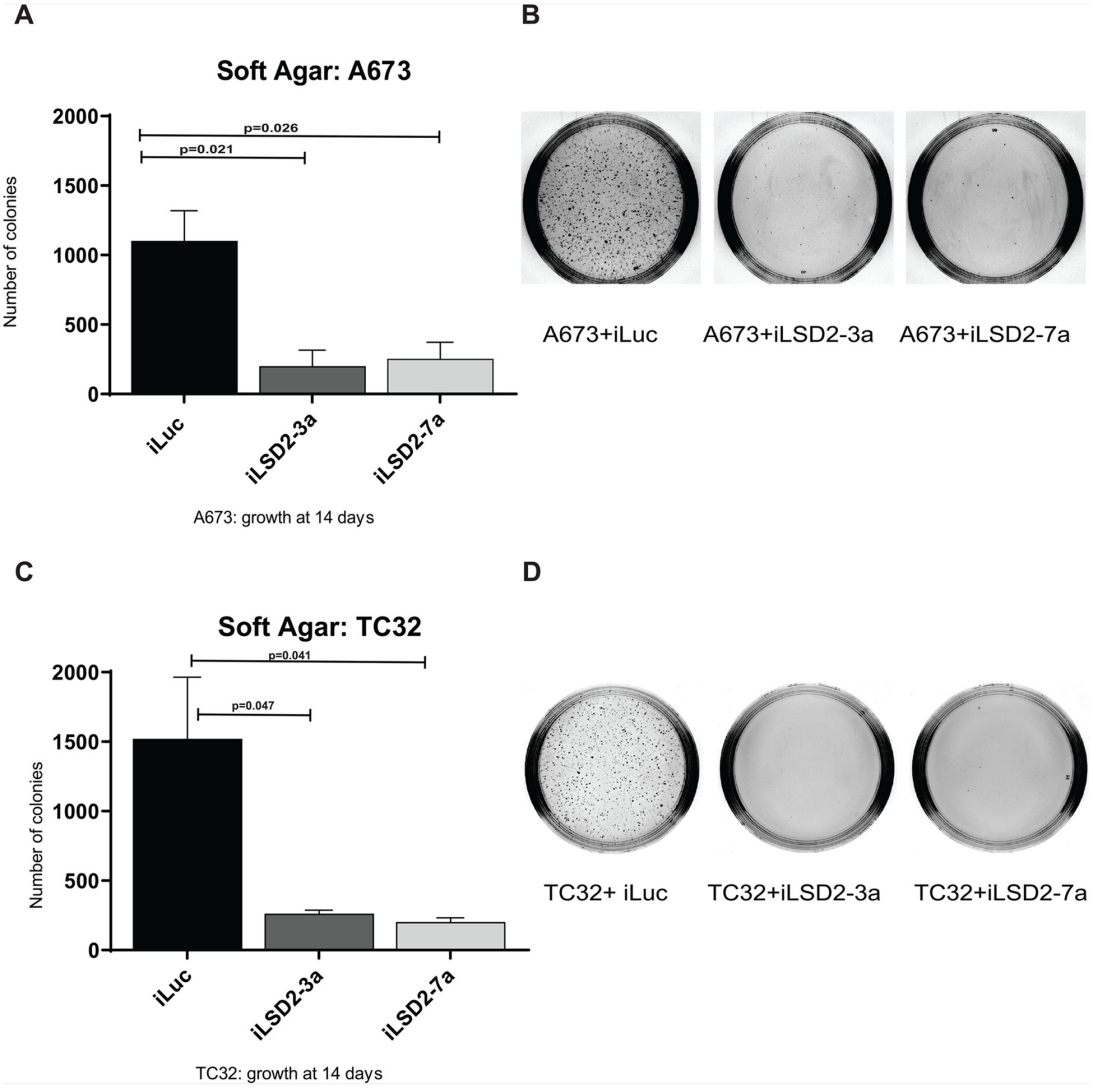
LSD2 knockdown significantly impairs the anchorage-independent growth capacity of Ewing sarcoma cells. Soft agar colony quantification of A673 **(A)** and TC32 **(C)** cells stably transduced with LSD2 or iLuc control shRNA constructs following puromycin selection. Data represents mean colony number ± SEM from 3 independent experiments. Representative agar images of A673 **(B)** and TC32 **(D)** cells treated as above, 14 days post seeding.

Following successful knockdown of LSD2, we assessed whether depletion of LSD2 has any impact on oncogenic transformation using soft agar assays to measure anchorage-independent growth. A significant 5.5- and 4.3-fold reduction in A673 colony number was noted after knockdown using iLSD2-3a and -7a, respectively, as compared to iLuc control (Figure 5A-5B). Similarly, for TC32 cells, a 5.8- and 7.5-fold reduction in TC32 colony number was noted after knockdown with iLSD2-3a and -7a, respectively (Figure 5C-5D). These data indicate that LSD2 is required for oncogenic transformation of Ewing sarcoma cells as measured by anchorage-independent growth.

### Transcriptional response to LSD2 knockdown in Ewing sarcoma cells

**Figure 6:**
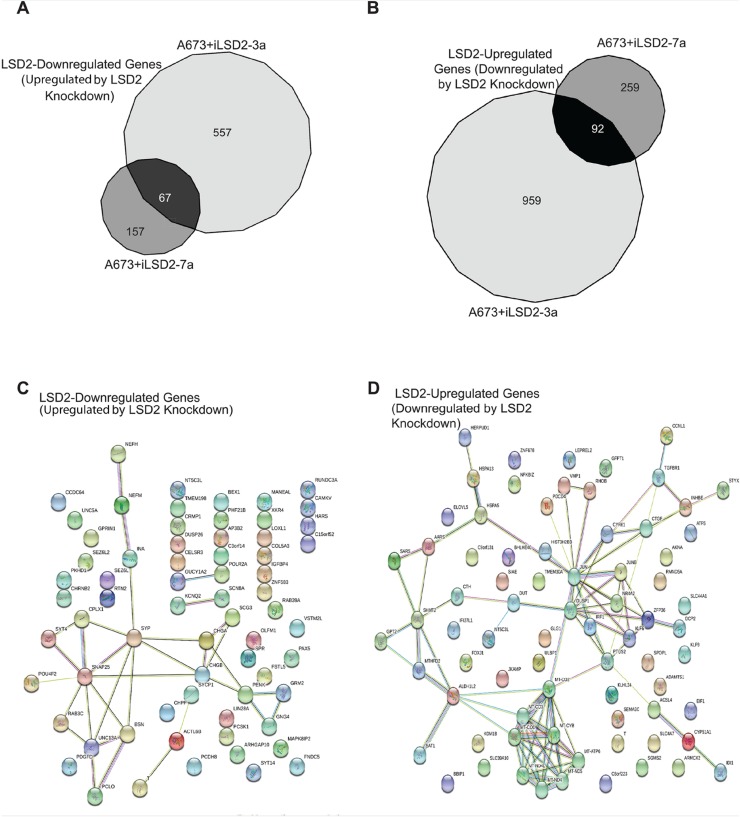
Core A673 LSD2 transcriptional profile. RNA seq Venn diagram analysis of genes significantly (>1.5 fold change) downregulated **(A)** and upregulated **(B)** by LSD2 in A673 cells. STRING pathway analysis of the core LSD2 downregulated (n=67) **(C)** and upregulated (n=92) **(D)** genes in A673 cells. The colored nodes represent the inputted proteins into STRING. The colors are automatically assigned by STRING and are used as a visual aid to show the proteins that have a functional relationship with each other (http://version10.string-db.org/help/faq/#are-the-colors-assigned-to-nodes-significant.) Empty nodes represent proteins of unknown structure, while filled nodes are proteins for which the 3D structure is known.

Because of the importance of LSD2 in oncogenic transformation of Ewing sarcoma cells, we next sought to understand the transcriptional function of this epigenetic regulator. We used RNA seq after LSD2 knockdown in A673 cells to identify genes specifically regulated by LSD2, and STRING pathway analysis was used to discern their functional relevance. For clarity, genes upregulated by knockdown of LSD2 will be referred to as “LSD2-downregulated” and genes downregulated after LSD2 knockdown will be referred to as “LSD2-upregulated”. Using a significance cut off of ≥1.5 fold change, knockdown of endogenous LSD2 with iLSD2-3a identified 624 LSD2-downregulated genes and 1051 LSD2-upregulated genes (Figure 6A-6B). Knockdown with iLSD2-7a identified 224 and 351 LSD2-downregulated and LSD2-upregulated genes, respectively (Figure 6A-6B). The greater number of modulated genes with iLSD2-3a suggests that this shRNA may in fact have had greater off-target effects when compared to iLSD2-7a. To identify a core group of LSD2 regulated genes, we reasoned that genes regulated by *both* iLSD2-3a and -7a are more likely to be on target. We used these core group of genes for subsequent analysis. Venn diagram overlap analysis revealed 67 LSD2-downregulated genes and 92 LSD2-upregulated genes common to both shRNA treatments (Figure 6A-6B).

To determine if there was significant pathway enrichment in our core subset of LSD2 regulated targets, STRING pathway analysis was performed (Figure 6C-6D) [20]. Pathway analysis of the core 67 LSD2-downregulated genes revealed 33 edges, or protein-protein associations (protein-protein interaction [PPI] enrichment p-value= 4.48 e-12; Figure 6C). Several biological process pathways were identified, including nervous system development, synaptic vesicle exocytosis, synaptic vesicle transport, and synaptic vesicle localization (Table 1). This is consistent with the suggested neural crest derivation of Ewing sarcoma cells [21]. STRING pathway analysis of the 92 common LSD2-upregulated genes revealed 83 protein-protein associations (PPI enrichment p-value <1.0e-16; Figure 6D). KEGG pathway analysis of these upregulated genes showed significant enrichment for metabolic pathways, oxidative phosphorylation, and Parkinson’s disease (Table 2). These genes play a role in small molecule metabolic processes and impact oxidative reductase activity. These data are consistent with the known high-metabolic rate described in Ewing sarcoma [22, 23].

**Table 1:** Pathway analysis for common LSD2-downregulated genes

Biological Process		
Pathway description	Count in gene set	False discovery rate
Synaptic vesicle exocytosis	7	5.1e-06
Synaptic vesicle transport	8	5.1e-06
Synaptic vesicle localization	8	5.1e-06
Synaptic transmission	12	0.00
Nervous system development	20	0.00
**Cellular Component**		
Presynaptic membrane	5	0.00
Synapse part	11	0.00
Neuron part	15	0.00
Neurofilament	3	0.00
Axon	9	0.00

**Table 2:** Pathway analysis for common LSD2-upregulated genes

Biological Process		
Pathway description	Count in gene set	False discovery rate
Small molecule metabolic process	26	2.24e-05
Organonitrogen compound metabolic process	21	0.00
Oxidative phosphorylation	6	0.00
Biosynthetic process	36	0.00
Organophosphate metabolic process	14	0.00
**Molecular Function**		
Oxidoreductase activity	13	0.00
**Cellular component**		
Respiratory chain complex	6	0.00
Respiratory chain	6	0.00
Organelle	60	0.00
Intracellular membrane-bounded organelle	55	0.00
Inner mitochondrial membrane protein complex	6	0.00
**KEGG Pathways**		
Metabolic pathways	21	1.82e-07
Oxidative phosphorylation	8	3.63e-06
Parkinson’s Disease	8	4.08e06
One carbon pool by folate	3	0.00
Cardiac muscle contraction	4	0.00

**Figure 7:**
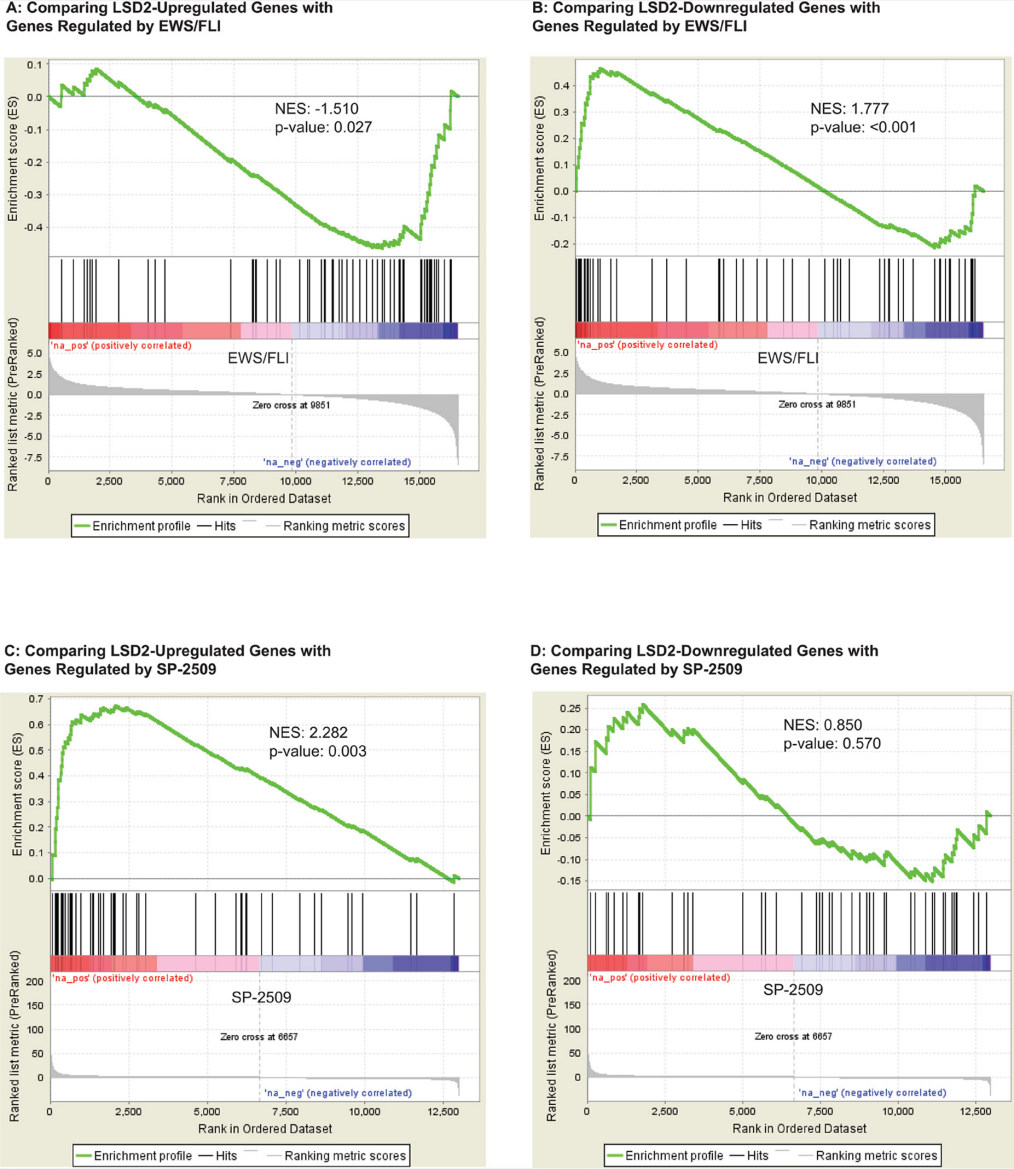
GSEA data supports a functional relationship between transcriptional genes regulated by LSD2 and EWS/FLI. **(A)** GSEA data comparing LSD2-upregulated genes with genes controlled by EWS/FLI. **(B)** GSEA data comparing LSD2-downregulated genes with ESW/FLI regulated genes. Genes up- **(C)** and downregulated **(D)** by LSD2 compared with genes regulated by SP-2509 drug treatment.

The primary oncogenic driver of Ewing sarcoma is EWS/FLI. Thus, we next wanted to understand how the transcriptional program mediated by LSD2 functionally relates to that of EWS/FLI. We observed that LSD2-upreglated genes correlated with genes repressed by EWS/FLI (NES= -1.510, p-value= 0.027) using gene set enrichment analysis (GSEA; Figure 7A). LSD2-repressed genes correlated with genes activated by EWS/FLI (NES= 1.777, p-value <0.001; Figure 7B). These data suggest that LSD2 may function as a transcription regulator of genes controlled by EWS/FLI.

The small molecule SP-2509 has been shown to globally disrupt EWS/FLI-mediated gene regulation via inhibition of LSD1 function [3, 5, 10]. Additionally, LSD2 expression impacts SP-2509 drug cytotoxicity [3]. Thus, we next asked whether LSD2-regulated genes correlate with the SP-2509 gene signature. GSEA revealed that LSD2-activated genes correlate with SP-2509 driven gene activation (NES= 2.282, p-value=0.003; Figure 7C). These genes are repressed by EWS/FLI and this corroborates the functional link between LSD2 activation at EWS/FLI repressed targets. In contrast, these data suggested that SP-2509 does not interfere with LSD2 activity at these targets, as drug treatment with SP-2509 does not inhibit LSD2-activated genes. No significant functional correlation was observed between SP-2509 driven gene downregulation and LSD2-mediated repression (Figure 7D). Taken together these data supported a model whereby LSD2 serves as a global transcriptional counterbalance for EWS/FLI, and this counterbalance may help cells fine tune the transcriptional output required to maintain malignant phenotypes. Ewing sarcoma is a transcriptionally driven cancer and decreasing expression of transcription regulators such as LSD2 may indeed then impact the oncogenic potential of the cells, as seen by decreased soft agar colony formation. However, while induction of LSD2 mRNA was previously shown to correlate with sensitivity to SP-2509, SP-2509 does not significantly impact LSD2’s role as a transcriptional regulator in Ewing sarcoma.

## DISCUSSION

Understanding the epigenetic misregulation of Ewing sarcoma is crucial for the development of novel therapies. Previous research has focused on studying the role of LSD1 in modulating the EWS/FLI transcriptional signature [3, 5, 10]. In this study, we investigated the function of LSD2 as an epigenetic regulator. We previously showed that reversible LSD1 inhibition with SP-2509 leads to the induction of apoptosis and disruption of the EWS/FLI transcriptional signature [5]. Furthermore, we also showed that LSD1 inhibition with SP-2509 decreases Ewing sarcoma cell viability through the engagement of the endoplasmic reticulum stress response [3]. Interestingly, knockdown of LSD2 expression led to decreased SP-2509 cytotoxicity in A673 cells, suggesting that the drug may impact LSD2 expression and/or function [3]. Together, these data warranted the study of the pharmocogenetic relationship between LSD2 and SP-2509 and the function of LSD2 in Ewing sarcomagenesis.

Based on the previous research, we hypothesized that SP-2509 inhibits LSD1 and LSD2 and that LSD2 mediates SP-2509 drug toxicity. Surprisingly however, our RNA seq and GSEA results implied that SP-2509 does not significantly impact the function of LSD2 as a transcriptional regulator in Ewing sarcoma. The data did show enrichment between genes activated by LSD2 and those activated by SP-2509 drug treatment, indicating that SP-2509 does not antagonize LSD2 function at these genes (Figure 7C). Moreover, there was no significant gene enrichment noted between the LSD2-downregulated genes and SP-2509 regulated genes (Figure 7D). Thus, we concluded that SP-2509 does not inhibit LSD2 function, suggesting that LSD2 regulates a unique cohort of genes independent of LSD1 and that SP-2509 displays specificity for LSD1. These data warrant the further study of LSD2 as a unique target for novel treatments.

The GSEA data did support a role for LSD2 as an important regulator of genes controlled by EWS/FLI (Figure 7A-7B). RNA seq and pathway analysis also suggested that LSD2 shares phenotypic similarity with EWS/FLI-regulated functions. We characterized the genes regulated by LSD2 using pathway analysis, which showed that LSD2 regulates several genes involved with cellular metabolic activity (Table 2). Protein-protein interactions that impact respiratory electron transport chain, mitochondrial ATP synthesis coupled electron transport, electron carrier activity, NADH dehydrogenase activity, and oxidoreductase activity were identified using STRING analysis (Figure 6D). Previous data suggested that Ewing sarcoma cells rely heavily on mitochondrial respiration and glycolysis [22]. Ewing sarcoma cells exhibited more glycolytic activity as compared to non-cancerous cells and metabolic inhibition decreased cancer cell proliferation and cell cycle progression [22]. Furthermore, another study demonstrated EWS/FLI’s crucial role in the regulation of metabolic reprogramming in Ewing sarcoma [23]. Metabolomic studies concluded that decreased expression of EWS/FLI increased respiratory and glycolytic functions [23]. Furthermore, our pathway analysis showed that LSD2 regulates metabolic function (Table 2). In conjunction with the previously reported data, we hypothesize that EWS/FLI may be able to function as a metabolic misregulator in part due to its interactions with LSD2. Thus, knockdown of LSD2 may lead to metabolic inhibition, which in turn impacts the oncogenic capacity of the Ewing sarcoma cells.

One hairpin showed a modest decrease in Ewing cell proliferation (iLSD2-3a), while the other did not (iLSD2-7a; Figure 4A-4B). Regardless of these minor differences, both hairpins demonstrated a crucial role of LSD2 for the oncogenic capacity of Ewing sarcoma cells. Significantly decreased anchorage-independent growth in soft agar was noted after the knockdown of endogenous LSD2 expression using both hairpins (Figure 5A-5D). The GSEA data further supported that LSD2 function correlates with transcriptional gene regulation by EWS/FLI (Figure 7A-7B).

Taken together, these data suggest that LSD2 plays a crucial role in Ewing sarcomagenesis. Ewing sarcoma is the second most common bone tumor in pediatric and young adult patients, and despite decades of research, the cure rates remain at about 75% for local disease and 20% for metastatic disease. Understanding mechanisms of epigenetic misregulation in Ewing sarcoma is important for our ability to further understand the basic biology of this aggressive malignancy. In addition, understanding epigenetics may prove to be extremely useful in developing novel targeted drug treatments. This report identifies a new opportunity by demonstrating the critical role of LSD2 in oncogenic transformation and its role in metabolic activities. Although there are currently no small molecule agents that specifically inhibit LSD2, our results lay the foundation for further investigation into LSD2 as a target for the treatment of Ewing sarcoma.

## MATERIALS AND METHODS

### Cell lines and compounds

Ewing sarcoma cells lines A673 and TC32 were obtained from American Type Culture Collection (Manassas, VA) and Dr. Timothy Triche (Children’s Hospital Los Angeles), respectively. Cell line identity was confirmed using STR profiling (Genetica LabCorp, USA) and cultured as previously described [3].

### Cloning

LSD2 was cloned from A673 cells using the NEBhifi DNA Gibson Assembly, gene specific primers, and PCR. LSD2 cDNA were inserted into the pMSCV-Blast vector (Addgene) in between sites BGLII and XHO1 and sequence verified.

### shRNAs

Knockdown of endogenous LSD2 was achieved through retroviral infection of cells with two targeted shRNAs: iLSD2-3a and iLSD2-7a [3]. A shRNA targeted against luciferase (iLuc) was used as a control. Sequences of hairpins and generation of shRNA constructs were as previously described [3]. Following infection, Ewing sarcoma cells were selected in puromycin (0.05-2mg/ml; Sigma-Aldrich) for 72 hours.

### Immunodetection

Western blot analysis was used to verify knockdown of LSD2 protein expression after retroviral infection with the two targeted shRNAs as previously described [3]. Antibodies used include LSD1 (Abcam, ab17721,1: 2000), LSD2 (Abcam, ab193080, 1:1500), and α-tubulin (Abcam, ab7291,1:2000) as described previously [3].

### qRT-PCR

Total RNA was extracted using the Qiagen RNAeasy kit with on-column DNase digestion according to the manufacturer’s instructions. qRT-PCR was performed on a Bio-Rad CFX Connect Real-Time System machine using iTaq Universal SYBR Green 1 Step Reaction Mix (Bio-Rad). Expression was normalized against the *RPL19* house keeper gene. PCR conditions and primer sequences were as described previously [3].

### Soft agar assays

Soft agar assays were utilized to examine the anchorage-independent growth capacity of A673 and TC32 cells following LSD2 knockdown as previously described, but modified by seeding 7500 to 12500 cells into duplicate 6cm plates [3]. Plates were incubated at 37˚C for 14 days. The number of colonies was quantified using Image J (Version 1.517) [3]. Three independent experiments were conducted for each condition.

### IncuCyte cell proliferation

The proliferative capacity of A673 and TC32 cells following LSD2 knockdown was assessed through IncuCyte (Essen) live cell imaging. Cells were seeded in triplicate into 96-well clear bottom micro-titer plates at varying concentrations (6000-10000 cells/well). Phase contrast images were collected every 3 hours for a maximum of 100 hours. Three independent experiments were completed for each cell line with confluency quantified using IncuCyte Zoom 2016A software as previously reported [3].

### RNA seq

Total RNA from A673 cells (iLuc and LSD2 knockdowns) was extracted using the Qiagen RNeasy Kit with on-column DNase digestion. RNA from two independent experiments was submitted to the Biomedical Genomics Core at Nationwide Children’s Hospital for paired-end RNA seq analysis with a read length of 150 base pairs. Quality control was done on raw sequences using FASTQC. No adapter sequences were detected and base quality sequences were good. RNA seq sequences were aligned to hg19 genome assembly using two pass STAR aligner. Annotation for transcripts was obtained from homo_sapiens.grch37.75.gtf (http://ftp.ensembl.org/pub/release-75/gtf/homo_sapiens/). All other parameters were set as default. After alignment using STAR, RSeQC was run to perform post-mapping quality control. Reads aligned to genomic features such as genes, exons, promoters and genomic bins were counted using feature counts from Subread package using default parameters. Differential gene analysis was done using DESeq2 and SARTools R packages. Significant differentially expressed features were identified with the criteria of a fold change of absolute value ≥1.5 and an adjusted p-value of <0.05 (5% FDR). Pathway analysis was conducted using STRING interaction pathway software [20].

### GSEA analysis

Gene set enrichment analysis (GSEA) was used to compare differential genes in different studies [24]. We used GSEA to compare the LSD2 RNA seq data with previous EWS/FLI and SP-2509 data sets [3, 10]. Significance was defined as the absolute value of the normalized enrichment score (NES) of ≥1.5 and p-value of <0.05.

### Statistical analysis

Soft agar, IncuCyte proliferation, qRT-PCR, and protein analysis data were interpreted using the mean of three independent experiments. P-values were calculated using Student t-test using Graph Pad Prism (Version 7). P-values of <0.05 were considered statistically significant.
